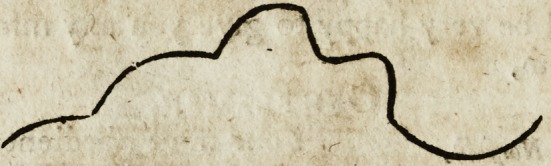# Cases of Dysphagia

**Published:** 1800-12

**Authors:** H. H. Helsham

**Affiliations:** Stoke Ferry, Norfolk


Cases of Dysphagia

THE

Medical and Phyfical Journal.

VOL. IV.] DECEMBER, 1800. [NO. XXII.

*To the Editors of the Medical and Phyfical Journal.*

GENTLEMEN,

IF you think the enclofed cafes deferving of a place in your ufeful Medical Journal, you will do me the favour of inferting them. If there are any queftions you wifh to afk concerning them, I fhall be very happy to give you any information in my power. I am,

GENTLEMEN,

Your moft obedient fervant,

H. H. HELSHAM, M. D.

*Stoke Ferry, Norfolk, Nov.* 6, 1800.

---

### *CASES of DYSPHAGIA.*

[ With Engravings. ]

### CASE I.

*An account of the diffection of a woman, aged* 65 *years, who had been fubject, for five years preceding her death, to a sickness and reaching, and a senfation of pain in swallowing in the track of the spine and œsophagus; in a letter to Dr.* HAMILTON, *of Lynn.*

DEAR SIR, *Stoke, April* 1, 1792.

Our patient, Mrs. Godman, lingered out till Thurfday laft. For the laft fortnight, I dare fay, fhe fcarce fwallowed any thing; and, for about that time, the fpitting and expectoration of mucus had ftopped. She was incapable of fpeaking for the laft four or five days, but retained her fenfes to the laft moment, though her pulfe had not been perceptible for two days.

We obtained leave to open the body, which my fon did yefterday.

Upon laying open the cavity of the abdomen, and feeling in the fituation of the ftomach, we were ftruck with the appearance of fome protuberant fwelling behind it; but as we wifhed firft to make a careful examination of the track of the œfophagus, he proceeded to the examination of the thorax and neck, and and diſſected the œſophagus and ſtomach carefully out from their ſituation; previous to which a probang was paſſed down into the ſtomach, which met with no impediment whatever.

When the ſtomach and œſophagus were diſſected out, he laid them open, and not the leaſt veſtige of diſeaſe, neither ſtricture, ſchirrus, ulceration, or even abraſion of the internal coat, appeared in either.

We now ſought for the enlargement which occaſioned the particular feel behind the ſtomach and viſcera.

This was no other than the ſpine itſelf, which formed a ridge from the neck to the laſt vertebræ of the back, and had more the feel of the ſpinal proceſſes of the back than the bodies of the vertebræ. Inſtead of the uſual form of the bodies of the vertebræ, as thus  the following was the form of them:

and the depth of the cavity, from the middle of the bodies of the vertebræ to the poſterior arch of the ribs, was at leaſt five inches.

The protuberance was greateſt about the ſeventh vertebræ of the back, where the œſophagus paſſes through the diaphragm.

The liver and all the viſcera appeared ſound, the omentum was very thick and large, but did not appear ſchirrous.

There were two biliary concretions in the gall bladder the ſize of a large nutmeg each.

How far will this particular form of the ſpine account for the ſymptoms ſhe had been ſubject to for five years?

### CASE II.

R. C. Eſq. aged 79, had been affected for twenty years paſt with Dyſphagia, which was ſuppoſed to have been firſt occaſioned by his eating a morſel of hot pudding, which, by excoriating the inſide of the œſophagus, might have given riſe to ſtricture, or fungus excreſcence in the part.

For the laſt two or three years of his life I had frequent opportunities of being at table with him, and from the obſervation I then made on his manner of ſwallowing, and what I ſaw during the week preceding his death, I could by no means think that either ſtricture or fungus ſufficiently accounted for it.

Excluſive of the difficulty of ſwallowing, in the firſt inſtance, he would ſome time afterwards (perhaps ten or fifteen minutes) regurgitate part of his food or drink back again, which ſeemed to have lodged ſomewhere in the paſſage.

Excepting

Excepting the difficulty of ſwallowing, he had enjoyed a very good ſtate of health, was of a fair, florid complexion, and rather inclining to corpulency.

In January, 1792, the difficulty of ſwallowing increaſed to ſuch a degree, as almoſt totally to prevent any nouriſhment being received into the ſtomach; and, for the laſt ten days of his life, he was ſupported by broth clyſters, and at length gradually ſunk from inanition.

During the laſt week of his life he would frequently, to appearance, ſwallow more than four ounces of liquid at a draught, which would remain ſometimes for five or ten minutes, and then be regurgitated back again.

Permiſſion being obtained to open the body; after removing the larynx, pharynx, trachea, and œſophagus, an enlargement or protuberance of the body of the fifth vertebra of the neck, which projected above half an inch over the body of the ſixth vertebra, appeared to be the original cauſe of the complaint, by producing the ſtricture in the œſophagus. This bony projection did not ſhow any mark of diſeaſe further than its enlargement.

The effort of ſwallowing had gradually produced a dilatation of the pharynx behind the œſophagus, and underneath the projecting body of the vertebræ, forming a ſac which would contain about four or five ounces, into which part of the food or drink eſcaped, and remained for ſome time, till by its irritation it produced an effort to reject it.

The annexed figures ſhew the parts after they had been prepared, and immerged in ſpirits.

Figure I. A back View of the Preparation.

*A.* The trachea.

*B.* The œſophagus.

*C.* The epiglottis.

*D.* The back part of the pharynx, cut open to ſhew

*E.* The ſtricture oppoſite to the protuberance of the vertebra.

*F.* The ſac, or bag, formed behind the œſophagus.

Figure II. A View of the Preparation on the left ſide.

*A.* The trachea.

*B.* The œſophagus.

*C.* The epiglottis.

*D.* The thyroid cartillage.

*E.* The cricoid cartilage.

*F.* The os hyoides.

*G.* The ſac, or bag, hanging down behind the œſophagus.

---

## Figures and Tables

**Figure f1:**
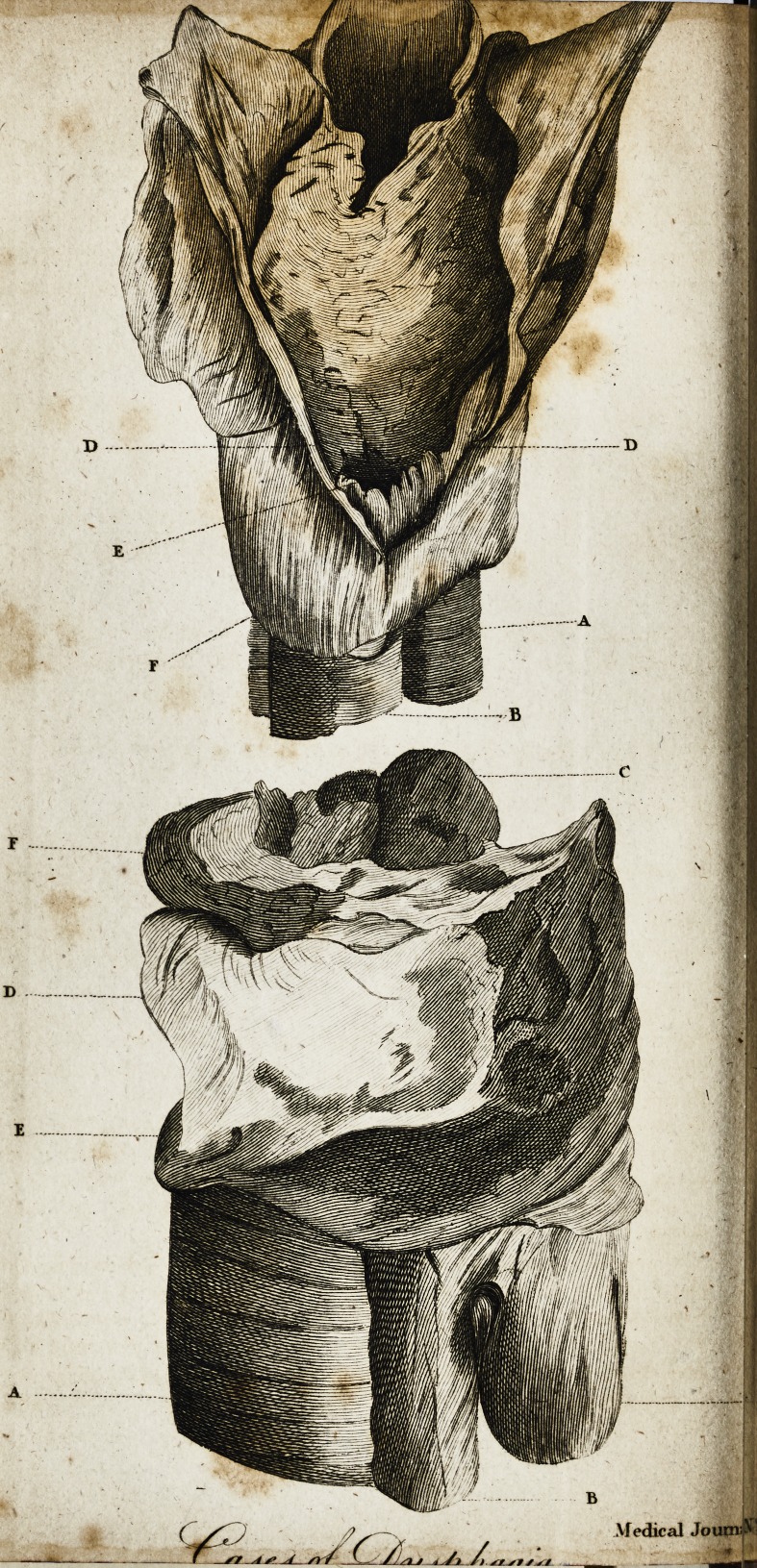


**Figure f2:**
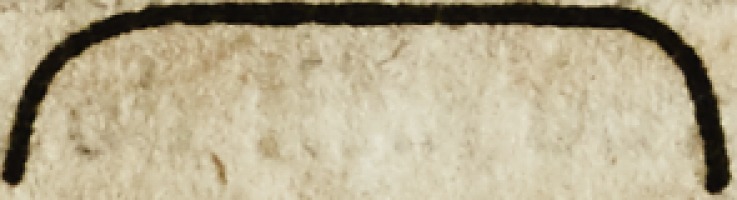


**Figure f3:**